# Modulation of Visual Contrast Perception Associated With Dorsal Attention Network Connectivity Assessed by Magnetoencephalography

**DOI:** 10.1002/hbm.70554

**Published:** 2026-05-24

**Authors:** Alfredo L. Sklar, Brian A. Coffman, Fran López‐Caballero, Dylan Seebold, Jenay Kocsis, Lauren Fowler, Hayley Rhorer, Jack Kavanagh, Dean F. Salisbury

**Affiliations:** ^1^ Department of Psychiatry University of Pittsburgh School of Medicine Pittsburgh Pennsylvania USA

**Keywords:** contrast response function, dorsal attention network, functional connectivity, magnetoencephalography, primary visual cortex

## Abstract

The impact of executive attention on visual cortical responses depends upon the contrast of input stimuli. Functional neuroimaging has difficulty capturing non‐linear gain modulations of the contrast response function (CRF) and dynamic communication within visual attention networks subserving it. The current study utilized magnetoencephalography (MEG) to examine gain modulation within primary visual cortex (V1) and its connectivity with regions of the dorsal attention network (DAN) during covert attention. Twenty‐five participants completed a spatial covert attention task including neutral and valid cues. MEG was recorded and eye position monitored throughout the task. The CRF and its relevant parameters were modeled using peak V1 evoked responses. Cue‐related alpha‐band desynchronization within contralateral V1 and event‐related spectral perturbations across DAN regions were assessed by wavelet analysis. Weighted phase‐lag index was used to examine DAN–V1 functional connectivity. Valid cue trials produced increased CRF maxima without a significant impact on mid‐saturation or baseline levels. Alpha desynchronization was observed between 10 and 12 Hz during cue presentation in V1. DAN—V1 functional connectivity, most robust within 10–12 Hz, was uniquely associated with larger asymptotic V1 response levels within this range. MEG recordings revealed a pattern of V1 response gain during covert attention associated with DAN—V1 connectivity, advancing our knowledge of this network's frequency‐specific influence over gain modulation within basic visual processing centers. These findings highlight the advantages of MEG for examining interactions between sensory and attentional gain properties of the human visual system, providing a comprehensive understanding of their underlying local and distributed network dynamics.

## Summary

- Magnetoencephalography was used to identify the impact of endogenous attention on V1 responses to stimuli of varying contrast and the communication with the DAN contributing to it.
- Attention produced enhancement of the maximal V1 response that was associated with synchronous communication between the DAN and V1 within the high alpha‐band oscillatory range.
- These results capture the modulation of stimulus‐driven visual activity and the mechanisms driving it on the anatomic and temporal scale of human brain network activity.

## Graphical abstract

Magnetoencephalography was used to identify the impact of spatial attention on primary visual cortical responses to stimuli of varying contrast levels. Enhancement of the maximal response of the visual cortex to high contrast stimuli was associated with synchronous communication between the dorsal attention network and visual cortex within the alpha‐band frequency range.

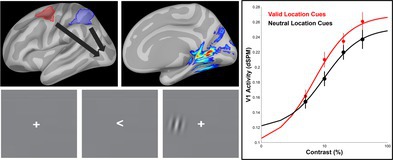

## 1 Introduction

Modulation of sensory inputs by attention is vital to the execution of coherent, goal‐directed behaviors. Understanding the complex local and long‐range neural interactions that bias processing of relevant items requires an appreciation for the non‐linear nature of stimulus‐evoked neurophysiological responses and their modulation by attention. The contrast response function (CRF) is a sigmoidal function characterizing response properties of the visual system to stimulus contrast including its rapid amplification at lower contrast levels and saturation at higher contrast levels. Parameters of the CRF including its maximal or saturation response at high contrast (*R*_max_), basal response level, and mid‐saturation point (*C*_50_) reflect distinct attributes of the visual system (Albrecht and Hamilton 1982; Albrecht et al. 2002). Understanding the modulation of these CRF parameters and the neural dynamics driving it provides insights into how attention shapes our experience of the external world.

Psychometric studies have identified distinct patterns of visual attentional gain depending on stimulus size and cue type (Herrmann et al. 2010). Response gain describes the multiplicative impact of attention across contrast level resulting in increased asymptotic responses (i.e., larger *R*_max_) whereas contrast gain reflects enhanced sensitivity to contrast characterized by a leftward shift of the CRF (i.e., lower *C*_50_). Single unit recordings and scalp electroencephalography (EEG) have validated these findings. Recordings of spike rates (Martınez‐Trujillo and Treue 2002; Williford and Maunsell 2006) and evoked voltage potentials isolated using EEG (Itthipuripat et al. 2014; Joon Kim et al. 2007) have identified response and contrast gain patterns without alterations in baseline visual response properties. These discoveries inform canonical models of neural computations such as normalization that explain mechanisms by which attention shapes information processing in the brain (Reynolds and Heeger 2009; Heuer and Britten 2002). However, these techniques lack spatial resolution, in the case of EEG, and distribution, in the case of single unit recordings, necessary to inform the inter‐regional network dynamics supporting it.

Historically, fMRI has been a vital tool for parsing distinct roles of executive networks in modulating visual processing given its exceptional spatial resolution and sensitivity to experimental manipulations of attention. Specifically, fMRI has been used to disentangle brain networks responsible for orienting and reorienting attention based on task and environmental demands. This work includes the isolation of distinct dorsal (DAN) and ventral (VAN) attention networks dedicated to the voluntary control of endogenous attention and its exogenous capture by salient stimuli, respectively (Chica et al. 2011; Tosoni et al. 2023; Vossel et al. 2014). Unfortunately, traditional fMRI block designs typically produce linear relationships between stimulus contrast and visual response (Ip et al. 2019; Marquardt et al. 2018), and examinations of attention effects emphasize an additive model (Buracas and Boynton 2007; Itthipuripat et al. 2019) characterized by a baseline shift where cortical activity is enhanced equally across all levels of stimulus contrast. Modulation of basal cortical activity suggests executive control uncoupled from external stimulus properties, limiting the ability of fMRI to elucidate interactions between sensory and attentional gain mechanisms.

Recent studies utilizing paradigms controlling for adaptation of the visual system to contrast suggest fMRI can capture non‐linear aspects of the visual response (Vinke et al. 2022) and multiplicative effects of attention (Foster and Ling 2022). However, these results are so far limited to feature‐based attention in which attention is directed to an attribute of a stimulus rather than its location. Furthermore, the BOLD signal remains suboptimal for identifying dynamic changes within sensory cortices and their reciprocal interactions with cognitive control centers occurring on the order of milliseconds. Despite advances in fMRI task‐based functional connectivity (Gonzalez‐Castillo and Bandettini 2018), neurophysiological methods afford superior temporal resolution necessary to appreciate dynamic, frequency‐specific network communications supporting attention. Alpha‐band oscillations are a marker of attentional suppression (Foxe and Snyder 2011), and alpha desynchronization within regions contralateral to the deployment of attention reflects the biasing of sensory processing within visual cortex (Bacigalupo and Luck 2019; Bagherzadeh et al. 2020). Furthermore, the alpha‐band has been proposed as a carrier frequency for inter‐regional phase synchronization between the frontal and parietal nodes of the DAN and visual cortex subserving attentional modulation (D'Andrea et al. 2019; Lobier et al. 2018).

The present study utilized magnetoencephalography (MEG) to examine CRF modulation by attention and the inter‐regional network interactions underlying it. In addition to superb spatio‐temporal resolution, MEG allows quantification of communication between cortical regions by assessing lag and consistency of oscillatory synchronization with minimal phase distortion by cortical volume currents (Baillet 2017), making it an ideal tool to investigate dynamic communication between disparate brain regions on a short time‐scale. MEG activity recorded during a covert spatial attention task was localized to primary visual cortex (V1) as well as both frontal and parietal nodes of the DAN in order to accomplish three goals: (1) define parameters of the CRF and their modulation by covert attention using peak V1 evoked responses, (2) identify attention‐mediated alpha‐band desynchronization within V1 and potential carrier frequencies for communication with the DAN using source‐localized time‐frequency analysis, and (3) examine patterns of connectivity between the DAN and V1 and their relationship to CRF modulation.

## 2 Methods

### 2.1 Participants

Twenty‐six individuals (13 female) between 18 and 35 years old were recruited for the study. Data from 1 participant was excluded due to lack of an identifiable V1 evoked response. All participants had ≤ 20/40 vision or corrected to that level, confirmed via Snellen chart. Participants were excluded if they had a history of concussion with sequelae, neurological comorbidity impacting neurophysiologic recordings, history of substance dependence, or < 9 years of education. All participants were paid for their participation and provided informed consent. All procedures were approved by the University of Pittsburgh IRB.

### 2.2 Visual Paradigm

Visual stimuli and tasks were developed using Presentation software (Neurobehavioral Systems; Berkley, CA). Stimuli were presented onto a back‐projected screen positioned 90 cm from the participant. The projector (PROPixx; VPixx Technologies), located outside the electromagnetically shielded room, presented images with a resolution of 1920 × 1080 dpi at a refresh rate of 60 Hz and a background display luminance of 50 cd/m^2^.

**FIGURE 1:**
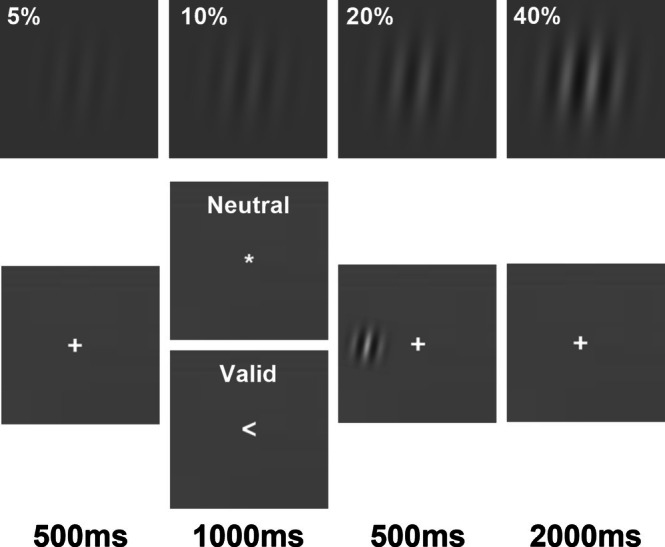
Example of Gabor stimuli of different contrast levels used (top) and depiction of an example trial from valid and neutral cue conditions (bottom).

The paradigm (Figure 1) consisted of a 2‐alternative forced choice task with two cue conditions (neutral vs. valid) manipulating covert spatial attention. Each trial began with a centrally presented fixation “+” (1° × 1°; 500 ms) followed by either a neutral (*) or valid (< or >) centrally presented cue (1° × 1°; 1000 ms) indicating the side of the screen of the to‐be‐presented target. The target was a gabor patch (sinusoidal grating of 0.4 cpd and diameter of 8° enveloped in a Gaussian window with sigma = 2.5°) located 10° lateral to the central cue (200 ms). Gabor patches were iso‐luminant with display background and presented at either 5%, 10%, 20%, or 40% Michelson luminance contrast and rotated ±7° off vertical. A centrally presented “+” then reappeared (1° × 1°; 2000 ms) during which participants indicated the direction of target rotation via button‐press.

Eye tracking (EyeLink 1000 Plus; SR Research, ON, Canada) was used to ensure fixation throughout the trial. Position data was sampled at 1000 Hz from the participants' right eye. Trials containing blinks or deviations in eye position > ±1.5° from central fixation along the horizontal axis were excluded. Data were collected from 100 trials for each combination of contrast level, target hemifield, and attention cue.

### 2.3 MEG Recording and Processing

MEG data were recorded using a 306‐channel MEGIN Neuromag TRIUX (Helsinki, Finland) system consisting of 306 sensors arranged into triplets, each with 2 orthogonal gradiometers and one magnetometer. Data were sampled at 1000 Hz (online bandpass filter = 0.1–330 Hz). Bipolar leads were placed at the outer canthi of both eyes to monitor horizontal eye movements, and single channel leads were placed below the left eye and on the left clavicle to monitor blinks and ECG, respectively. A 3D digitizer (ISOTRAK; Polhemus Inc., Colchester, VT) was used to record the location of five head positioning indicator (HPI) coils that were placed on the scalp and used to monitor head movements throughout the scan.

Initial pre‐processing steps were conducted to remove artifacts. Neuromag MaxFilter software (http://imaging.mrc‐cbu.cam.ac.uk/meg/Maxfilter_V2.2) was used to correct for head motion and apply the temporal extension of the Signal Space Separation (Taulu and Hari 2009) to remove electromagnetic noise originating from outside the MEG helmet. Data were imported into EEGLAB (Delorme and Makeig 2004) where a 0.5 Hz high‐pass filter was applied, bad channels and corrupted segments of data were identified, and an adaptive mixture independent component analysis (AMICA) was used to isolate and remove physiologic artifacts (Sklar et al. 2024; Klug et al. 2024).

Additional processing steps were conducted using Brainstorm (Tadel et al. 2011). A low‐pass 40 Hz filter was applied and data were segmented around target (−100–500 ms) and cue (−500–1500 ms) events. Epochs were baseline normalized. Trials with data exceeding ±5 fT for magnetometers and ±5 fT/cm for gradiometers or with saccades exceeding ±1.5° from fixation following the event of interest were excluded.

### 2.4 Source Localization

Structural MRI scans were obtained from each participant to provide accurate cortical localization of MEG sensor activity. A Siemens Magnetom Prisma Fit 3 T system was used to acquire T1 [TR/TE/TI = 2400/2.22/1000 ms, flip angle = 7°, FOV = 256 × 240 mm, voxel size = 0.8 mm3, 208 slices, GRAPPA acceleration factor = 2] and T2 [TR = 3200 ms, TE = 563 ms, FOV = 256 × 240, voxel size = 0.8 mm^3^, 208 slices] weighted images as well as a 10 min resting‐state fMRI scan [TR = 800 ms, TE = 37 ms, multiband factor = 8, flip angle = 52°, FOV = 208 × 208 mm, voxel size = 2.0 mm^3^, 72 slices]. MRI scans were processed according to Human Connectome Project (HCP) pipelines (Glasser et al. 2013) to obtain HCP cortical parcellations of the cortical surface for each participant using multivariate mapping from resting state networks, sulcal/gyral markers, and T1/T2 ratios.

MEG sensor data was registered to each participant's MRI scan by aligning the three fiducial points digitized in the participant during MEG acquisition (nasion and left‐ and right‐ear pre‐auricular points) with their correspondent points manually marked in the MRI image. A forward solution was modeled as overlapping spheres. A noise covariance matrix was calculated from the baseline window of all trials and used to pre‐whiten data to bring magnetometers and gradiometers into comparable scales. Source activity, derived using both magnetometers and gradiometers, was constrained to the gray/white matter boundary segmented using Freesurfer (http://www.surfer.nmr.mgh.harvard.edu) and tessellated into an icosahedron mesh with 75,000 vertices across both hemispheres. Source activity was estimated using the minimum norm estimation with an orientation constraint of 0 and depth‐weighting applied. For measurement of evoked visual responses, a dSPM statistic was calculated at each vertex, normalizing the current estimate to the pre‐stimulus baseline covariance.

### 2.5 Contrast Response Modeling

**FIGURE 2:**
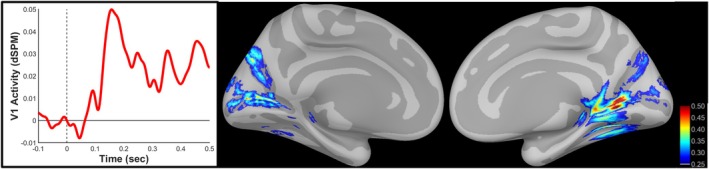
Time‐course of evoked V1 activity averaged across target contrast and cue condition. Cortical activity averaged over peak (145–175 ms) V1 target‐evoked activity window morphed onto the FreeSurfer template brain.

The CRF was modeled using source‐localized V1 activity. V1 was selected for the current investigation given the diminished contrast sensitivity of higher‐order visual processing centers (Avidan et al. 2002; Buracas and Boynton 2007) and the presence of robust attention‐related modulation of V1 activity beginning as early as 50 ms post‐stimulus (Kelly et al. 2008; Poghosyan and Ioannides 2008). Figure 2 depicts the time‐course of V1 responses to gabor targets presented in the contralateral hemifield averaged across contrast level and cue condition along with cortical localization of MEG activity averaged over peak V1 activity (145–175 ms) morphed onto the FreeSurfer template surface. Evoked neurophysiological signals including event‐related potentials (Itthipuripat et al. 2019) and steady‐state potentials (Ash et al. 2024; Di Russo et al. 2001) as well as evoked signals localized to visual cortex using MEG (Hall et al. 2005) have successfully been used to model the CRF. Peak V1 responses to gabor stimuli presented in the contralateral hemifield at each contrast level were used to model the CRF according to the Naka‐Rushton equation (Naka and Rushton 1966):

$$R\left(C\right)=R_{\max}\;\left(C^{n}/\left(C^{n}+{C_{50}}^{n}\right)\right)+R_{0}$$

where *C* refers to the input stimulus contrast, *R*_max_ refers to the asymptotic saturation response, *R*_0_ refers to the baseline response level, *C*_50_ refers to the mid‐saturation contrast level, and *n* refers to the slope of the function at *C*_50_. Values for *R*_max_, *n*, *C*_50_, and *R*_0_ were bounded between 0–2, 0–10, 0–100, and 0–2, respectively. Given the possibility of *R*_max_ exceeding *R*(100), this value was calculated as *R*_max_ = *R*(100) – *R*_0_ and *C*_50_ as the contrast level achieving one‐half *R*_max_ in accordance with previous literature (Itthipuripat et al. 2019).

### 2.6 Time‐Frequency Analysis

Time‐frequency analyses were performed on source‐localized activity during the cue interval within V1 as well as HCP‐derived (Allan et al. 2019) frontal (6a, FEF) and parietal (7 AM, 7PC, AIP, LIPd, LIPv, VIP) lobe regions of the DAN. A Morlet wavelet transformation (Tallon‐Baudry and Bertrand 1999) (FWHM = 3 s@*f* = 1 Hz; 1 Hz steps from 1 to 30 Hz) was applied to individual trial data during the valid cue condition. Individual trials were baseline corrected (−300–0 ms pre‐cue) and averaged to obtain a measure of total power. A wavelet transformation was also applied to the trial averaged data to obtain evoked power which was subtracted from total power to derive the measure of induced power for each participant. While this subtraction method assumes a temporally stable signal and risks introducing spurious power modulation, it also mitigates distortions of event‐related induced power by phase‐locked evoked responses and represents a standard practice in the field. Consistent with previous literature (Bacigalupo and Luck 2019), alpha‐band suppression within V1 was isolated by subtracting induced power time‐frequency plots obtained from V1 during trials where attention was deployed ipsilaterally (e.g., left hemisphere V1 during attend left trials) from contralateral ones (e.g., left hemisphere V1 during attend right trials) (Figure 4A). Time‐frequency plots for left and right hemisphere prior to subtraction are presented in Figure S1.

### 2.7 DAN‐V1 Connectivity Analysis

Time‐frequency power within DAN nodes during the cue phase is depicted in Figure 4B and broadband activity in Figure S2. Functional connectivity between V1 contralateral to the attended hemifield during cued trials and bilateral frontal and parietal nodes of the DAN was assessed during the cue phase (100–1000 ms post‐cue) using the weighted phase lag index (wPLI) approach (Vinck et al. 2011). The phase lag index measures oscillatory synchronization between two signals by quantifying the asymmetry in the distribution of instantaneous phase differences between them (Stam et al. 2007). This distribution is weighted by the imaginary component of the cross‐spectrum to obtain the wPLI, a measure of connectivity less sensitive to zero‐phase lag relationships indicative of field spread between signals. Source activity was band‐pass filtered (10–12 Hz) based on previous work identifying the high alpha as the dominant carrier frequency between attention and visual networks (D'Andrea et al. 2019; Lobier et al. 2018) as well as current data showing robust cue‐related suppression of V1 power (Figure 4A) and a prominent signal within the DAN at this frequency range across the cue interval (Figure 4B). A Hilbert transform was applied to extract phase values used for calculation of wPLI, a unitless measure ranging from 0 to 1. To establish spectral selectivity in the current dataset, the same analytic pipeline was also carried out for theta (4–7 Hz), low‐alpha (8–9 Hz), and beta (13–30 Hz) frequency bands (Niedermeyer and da Silva 2005). Connectivity between V1 and primary auditory cortex (A1) was also assessed to serve as a non‐attention related comparison control.

### 2.8 Data Analysis

Paired‐samples *t*‐tests were used to assess the impact of covert attention on the four parameters of the CRF derived from V1 activity. Due to ceiling effects, the CRF based on task performance could not be modeled using the Naka‐Rushton equation. Rather, 2 (cue: valid/neutral) × 4 (contrast: 5%/10%/20%/40%) repeated‐measures ANOVAs were conducted using accuracy (ACC) and response time (RT) data. To identify the presence of cue‐induced V1 alpha‐band desynchronization as well as the frequency‐ and time‐window specificity of this effect, power values during the cue interval (0–1000 ms post‐cue) across the broader alpha‐band frequency range (8–12 Hz) obtained from contralateral–ipsilateral V1 time‐frequency plots were subjected to parametric testing with FDR correction (*q* < 0.1) for time and frequency. While previous work suggests bilateral engagement of the DAN during covert attention (Maechler et al. 2025; Mayrhofer et al. 2019; Tosoni et al. 2023), there exist notable exceptions arguing a contralateral preference (Corbetta and Shulman 2011). Similarly, while previous work suggests connectivity between the DAN and visual cortex occurring primarily within the high alpha‐band (D'Andrea et al. 2019; Lobier et al. 2018), a consideration of spectral selectivity was warranted for the current connectivity analysis. Therefore, wPLI between bilateral frontal and parietal regions of the DAN and V1 contralateral to the attended hemifield was calculated over the post‐cue time window and assessed using a 2 (node: frontal/parietal) × 4 (frequency: theta/low‐alpha/high alpha/beta) × 2 (attention hemifield: left/right) × 2 (DAN hemisphere: contralateral/ipsilateral) repeated‐measures ANOVA. Partial correlations were used to examine the relationships between V1 alpha‐band suppression (log‐transformed values) and DAN‐V1 connectivity with V1 CRF parameters, disentangling the independent contribution of each to the modulation of visual contrast perception.

## 3 Results

### 3.1 Task Performance

Task performance (ACC and RT) data (M ± SD) are presented in Table 1. An interaction between cue condition and stimulus contrast was observed for both ACC (*F*_3,72_ = 5.84, *p* = 0.001) and RT (*F*_3,72_ = 2.99, *p* = 0.04). Participants responded more accurately to stimuli of 5% (*t*_23_ = 4.18, *p* < 0.001, *d* = 0.85) and 20% (*t*_23_ = 2.50, *p* = 0.02, *d* = 0.51) contrast stimuli during valid compared to neutral cue trials, but this was not the case 10% or 40% contrast stimuli (*p's* > 0.05). Responses to 5% (*t*_23_ = −3.34, *p* = 0.003, *d* = 0.68) and 10% (*t*_23_ = −2.93, *p* = 0.008, *d* = 0.66) stimuli were more rapid during valid compared to neutral cue trials, but not for 20% and 40% stimuli (*p's* > 0.05). In addition, main effects of ACC and RT were also present. Across contrast levels, responses were more accurate (*F*_1,24_ = 15.01, *p* < 0.001, *d* = 0.81) and rapid (*F*_1,24_ = 8.57, *p* < 0.007, *d* = 0.55) during valid cue trials. Across cue conditions, while responses to 5% contrast stimuli were less accurate compared to all other contrast levels (10%: *t*_23_ = −4.95, *p* < 0.001, *d* = 1.0; 20%: *t*_23_ = −4.62, *p* < 0.001, *d* = 0.94; 40%: *t*_23_ = −4.57, *p* < 0.001, *d* = 0.93), no differences were observed amongst these higher contrast stimuli (*p's* > 0.05). Responses were also slower to 5% (10%: *t*_23_ = 9.94, *p* < 0.001, *d* = 2.0; 20%: *t*_23_ = 10.49, *p* < 0.001, *d* = 2.1; 40%: *t*_23_ = 10.59, *p* < 0.001, *d* = 2.2), 10% (20%: *t*_23_ = 6.29, *p* < 0.001, *d* = 1.3; 40%: *t*_23_ = 7.68, *p* < 0.001, *d* = 1.6), and 20% (40%: *t*_23_ = 5.41, *p* < 0.001, *d* = 1.1) stimuli relative to stimuli of higher contrast levels.

**TABLE 1:** Task accuracy and response time data (M ± SD).

	5%	10%	20%	40%
Accuracy (%)				
Valid cue	95.2 ± 3.5	96.4 ± 3.0	97.1 ± 2.1	97.1 ± 2.1
Neutral cue	91.9 ± 6.4	95.6 ± 3.4	95.7 ± 2.1	96.2 ± 3.0
Response time (ms)				
Valid cue	565.2 ± 100.8	528.5 ± 86.3	517.9 ± 84.4	505.9 ± 83.6
Neutral cue	587.7 ± 93.1	544.3 ± 79.7	525.6 ± 77.5	517.9 ± 73.9

### 3.2 V1 CRF Modulation

**FIGURE 3:**
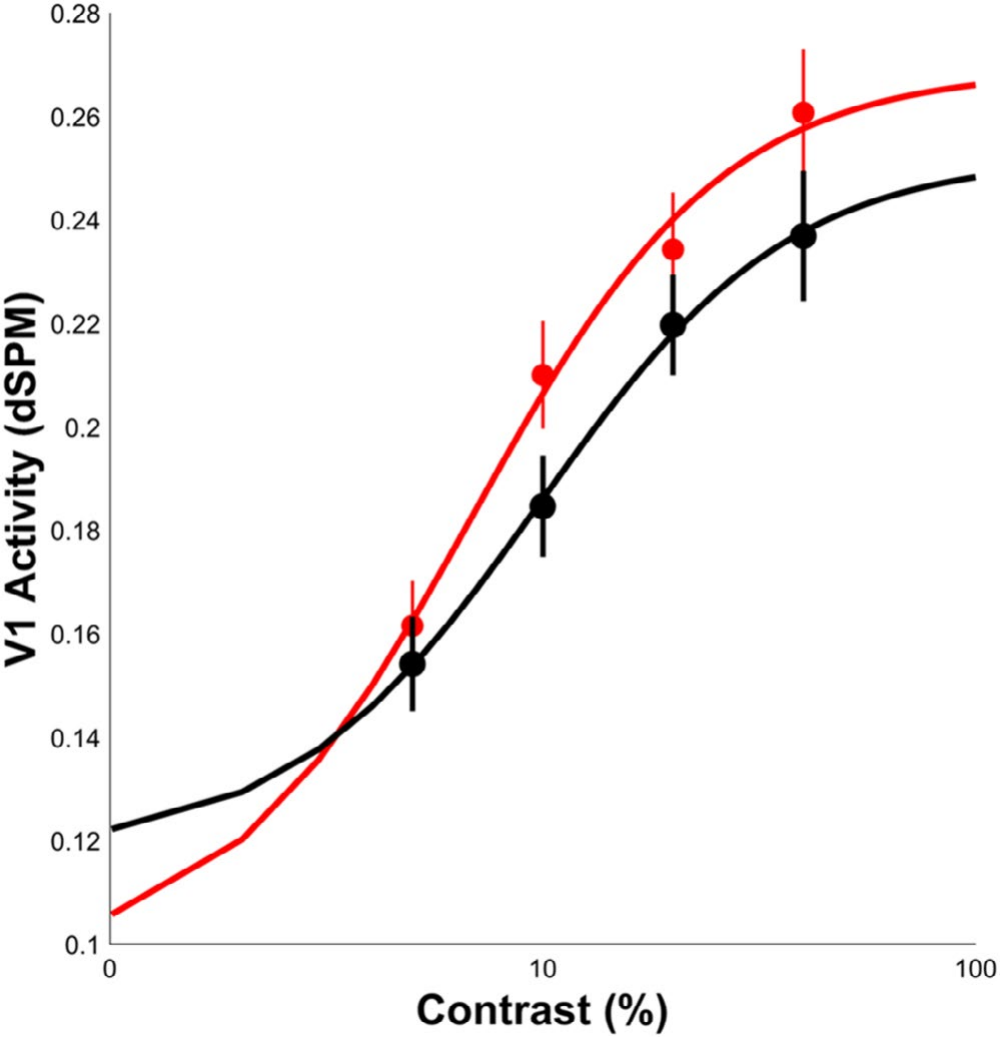
V1 CRFs for valid (red) and neutral (black) cue trials modeled using the Naka‐Rushton equation.

V1‐derived CRFs for both cue conditions are depicted in Figure 3 and values (M ± SD) for *R*_max_, *C*_50_, *n*, and *R*_0_ are presented in Table 2. Valid‐cue trials produced significantly larger *R*_max_ values compared to neutral cue trials (*t*_24_ = 3.27; *p* = 0.003, *d* = 0.68). No significant effects of cue condition were observed on *C*_50_ (*p* = 0.45), *n* (*p* = 0.26), or *R*_0_ (*p* = 0.10).

**TABLE 2:** CRF parameters modeled from V1 data acquired during valid and neutral cue trials.

	*R*_max_	*C*_50_	*n*	*R*_0_
Valid	0.219 ± 0.09	9.08 ± 10.6	2.59 ± 2.96	0.069 ± 0.05
Neutral	0.151 ± 0.09	11.37 ± 9.8	3.67 ± 3.6	0.098 ± 0.09

### 3.3 V1 Alpha‐Band Desynchronization

**FIGURE 4:**
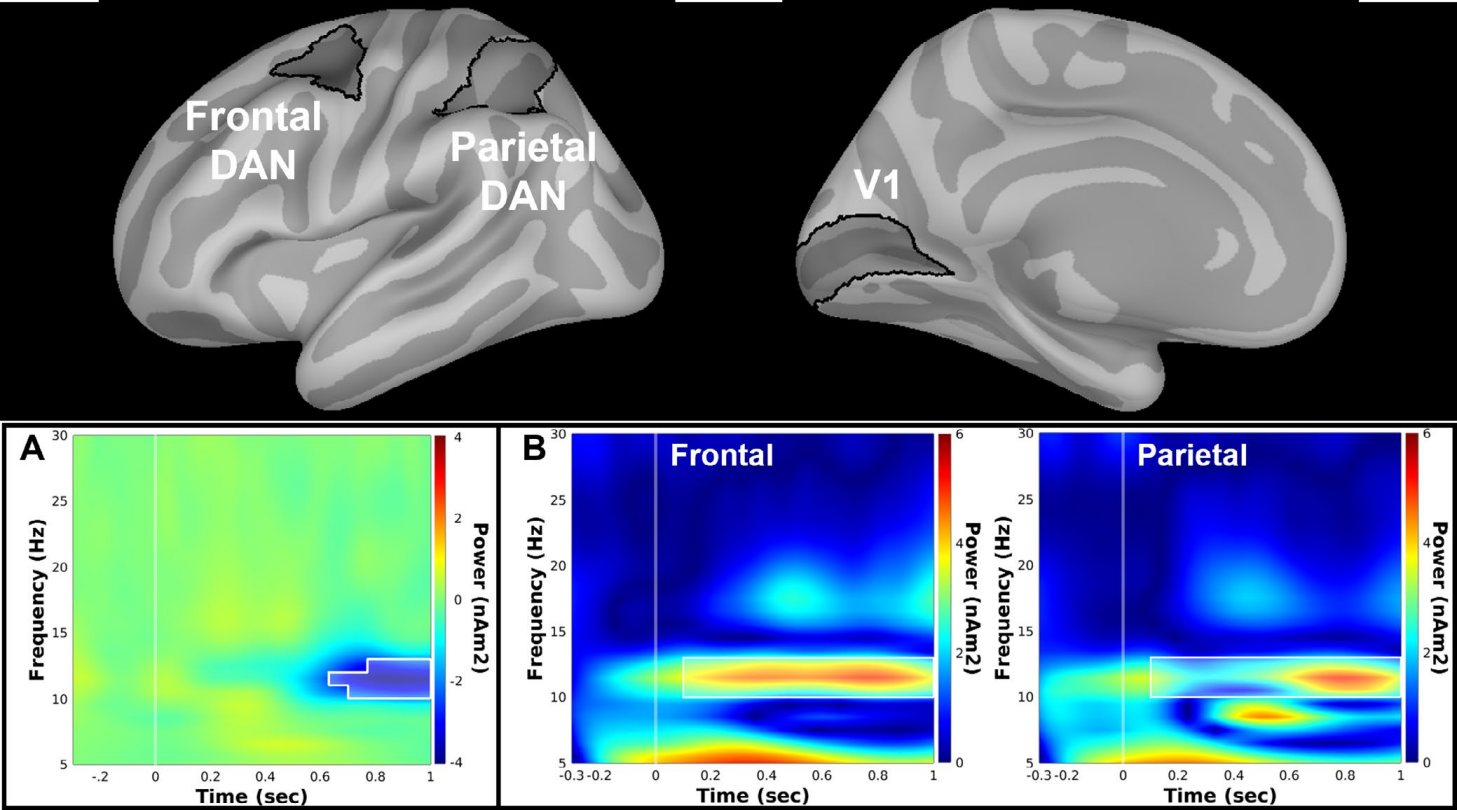
V1 contralateral minus ipsilateral time‐frequency spectrum (A) with white outline depicting time‐frequency clusters surviving FDR correction. Time‐frequency spectra for the frontal and parietal regions of the DAN (B) with white outline depicting window over which connectivity with V1 was computed.

Time‐frequency plots for left and right hemisphere V1 activity following left and right visual field cues prior to subtraction are depicted in Figure S1 and merged contralateral–ipsilateral V1 plots are depicted in Figure 4A. Significant suppression of alpha‐band power contralateral to attended hemifield was observed within V1 between 10 and 12 Hz starting at 636 ms post‐cue and continuing until the end of the cue interval. Alpha‐band suppression values were extracted from this time‐frequency window for each participant for purposes of correlation analyses.

### 3.4 DAN‐V1 Connectivity

Connectivity data are presented in Table 3. There was a significant effect of DAN node (*F*_*1,23*_ = 20.09, *p* < 0.001) with larger wPLI values observed between parietal compared to frontal regions of the network and V1 contralateral to the attended hemifield. A significant effect of frequency was also present (*F*_*3,69*_ = 24.30, *p* < 0.001). Connectivity was most robust within the high alpha‐band (theta: *t*_24_ = 5.15, *p* < 0.001, *d* = 1.0; low‐alpha: *t*_24_ = 3.31, *p* = 0.003, *d* = 1.0; beta: *t*_24_ = 6.35, *p* < 0.001, *d* = 1.3), followed by low‐alpha (theta: *t*_24_ = 3.20, *p* = 0.004, *d* = 0.64; beta: *t*_24_ = 7.28, *p* < 0.001, *d* = 1.1), theta (beta: *t*_24_ = 5.69, *p* < 0.001, *d* = 1.5), and beta bands. While there was an interaction between frequency and attended hemifield (*F*_*3,69*_ = 3.00, *p* = 0.04), no differences were observed between attend left versus right trials within each frequency band (*p's* > 0.1). An interaction was also present between frequency and DAN node (*F*_*3,69*_ = 8.23, *p* < 0.001) with the parietal node exhibiting stronger V1 connectivity within each frequency band (theta: *t*_24_ = 2.52, *p* = 0.02, *d* = 0.80; low‐alpha: *t*_24_ = 3.08, *p* = 0.005, *d* = 0.50; high alpha: *t*_24_ = 3.91, *p* < 0.001, *d* = 0.62) relative to the frontal node except beta (*p* = 0.08). There were no differences in connectivity between left and right attended hemifield trials (*p* = 0.24) or contralateral and ipsilateral DAN hemispheres (*p* = 0.18). No interactions between any other factors were present (*p's* > 0.05). Comparison between DAN‐V1 and A1‐V1 connectivity revealed significantly larger high alpha‐band wPLI values for the parietal DAN node (*t*_25_ = 4.05, *p* < 0.001, *d* = 0.80) and a trend toward larger values for the frontal DAN node (*t*_25_ = 1.91, *p* = 0.07, *d* = 0.38).

**TABLE 3:** wPLI values depicting synchronization between the DAN and V1 across frequency bands.

	Theta	Low Alpha	High Alpha	Beta
DAN Frontal‐V1	0864 ± 0.01	0.0928 ± 0.01	0.1078 ± 0.02	0.0802 ± 0.01
DAN Parietal‐V1	0.0899 ± 0.01	0.1013 ± 0.03	0.1271 ± 0.03	0.0809 ± 0.01

### 3.5 Correlation Analyses

**FIGURE 5:**
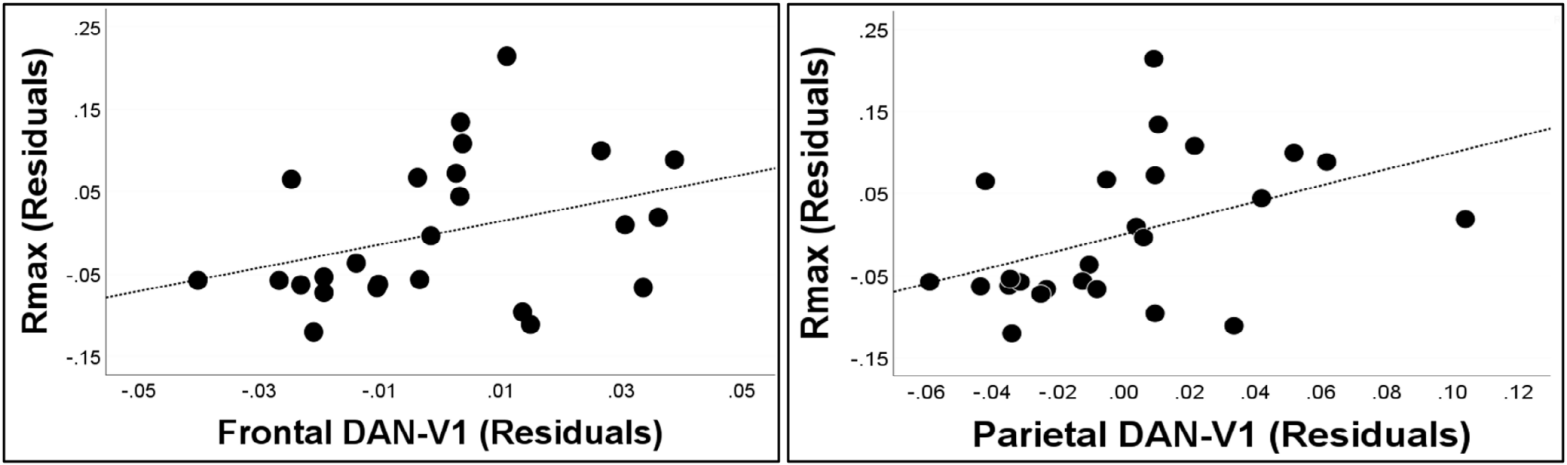
Partial regression plots depicting the relationship between residuals frontal and parietal DAN—V1 functional connectivity and V1 *R*_max_ during valid cue trials, controlling for V1 alpha‐band desynchronization.

Because the effect of attention did not differ between hemispheres or stimulus visual field presentation, partial correlations were run for frontal and parietal nodes across these factors. V1 *R*_max_ values were positively correlated with high alpha‐band connectivity between V1 and both frontal (*rho* = 0.53; *p* = 0.008) and parietal (*rho* = 0.56; *p* = 0.004) DAN regions after controlling for the influence of V1 alpha‐band desynchronization. Partial regression plots depicting these relationships are depicted in Figure 5. *R*_max_ was not associated with V1 alpha‐band suppression after controlling for frontal and parietal DAN‐V1 connectivity (*p* = 0.50). Of note, *R*_max_ was not associated with frontal or parietal DAN‐V1 connectivity in any other frequency bands (*p's* > 0.1) or with A1‐V1 connectivity within the high alpha‐band (*p* = 0.69) (Figure S3).

## 4 Discussion

The current study sought to capture the non‐linear qualities of the visual sensory system and its interactions with an executive attention network responsible for the modulation of its contrast‐dependent response properties. Utilizing MEG recordings, we identified a multiplicative response gain of primary visual cortex activity by covert attention. Modulation of stimulus‐evoked V1 activity was driven by frequency‐specific, oscillatory synchronization with frontal and parietal regions of the DAN. These findings advance our knowledge of sensory gain control within the visual system and provide critical insights into dynamic communications across the broader cognitive control network shaping our perception of the external world, informed by internal goals.

Contrary to previous functional neuroimaging studies describing a linear response to increasing contrast (Ip et al. 2019; Marquardt et al. 2018), MEG activity localized to V1 revealed a non‐linear CRF with a steep rise in activity at lower contrast levels that begins to saturate quickly, replicating previous studies using direct measures of neural activity to characterize response properties of visual cortex (Albrecht and Hamilton 1982; Albrecht et al. 2002; Contreras and Palmer 2003). Importantly, source‐localized V1 activity during our covert spatial attention task reveals a pattern of response gain with attention enhancing activity multiplicatively across stimulus contrast levels. The significant increase in asymptotic response levels with negligible impacts on basal activity stands in contrast to additive models of attentional gain modulation typically observed in fMRI studies in which enhanced V1 activity is observed across stimulus contrast levels (Buracas and Boynton 2007; Itthipuripat et al. 2019). These results highlight the distinction between functional neuroimaging studies identifying attention‐related shifts in basal visual cortical activation and neurophysiological studies capturing interactions between sensory and executive gain mechanisms to shape our perception. The observed multiplicative gain pattern lacking significant modulation of the CRF R_0_ parameter (lower *R*_0_ values were observed on valid trials) is also consistent with most human psychometric performance studies (Cameron et al. 2002). One possibility for the discrepancy between imaging modalities is that covert attention modulates non‐neuronal factors driving the BOLD response such as regional blood flow patterns or oxygen metabolism.

The observed increase in asymptotic response levels with negligible impacts on the mid‐saturation point is consistent with predictions of the NMA for tasks utilizing large target stimuli and a narrow field of attention. According to the NMA, stimulus inputs drive both excitatory responses as well as suppressive influences from surrounding neural populations that serve to normalize the overall output (Carandini and Heeger 2012). When the stimulus is large and attention is narrowly focused, attention enhances the entire excitatory field but only a portion of the suppressive field resulting in a vertical scaling of the CRF proportional to stimulus drive (Herrmann et al. 2010; Ni et al. 2012; Reynolds and Heeger 2009). Given the relatively large target stimuli and a cue that was 100% valid with consistent target locations across trials allowing participants to deploy a more narrow field of attention, we would predict the observed response gain pattern with modulation of the visual response increasing with stimulus contrast level. While task performance data, particularly accuracy, appears to contradict the response gain account with greater V1 response modulation at lower contrast levels, the presence of a ceiling effect limited our ability to assess non‐linear scaling of responses by contrast level and their modulation by attention. Accuracy was > 95% at all contrast levels across conditions except for the 5% contrast target during the neutral cue (91.9%). Not surprisingly, a significant cue effect was observed for this target contrast level.

Critical to understanding the modulation of visual perceptual responses by attention is examining the interregional network dynamics occurring prior to stimulus presentation while attention is being deployed. Building upon previous human neurophysiological studies, this investigation leveraged the spatiotemporal resolution of MEG to identify localized, frequency‐specific network dynamics supporting it. V1 alpha‐band desynchronization, frequently implicated in deployment of covert attention following simple and complex cues (Bacigalupo and Luck 2019; Bagherzadeh et al. 2020; de Vries et al. 2021), was observed within the high alpha range (10–12 Hz) during cue processing. In support of previous literature implicating the DAN in top‐down attentional control (Corbetta and Shulman 2002; Vossel et al. 2014), a prominent cue‐related high alpha‐band response was observed within frontal and parietal regions of this network and spectral connectivity between DAN regions and V1 within this frequency range was associated with *R*_max_ values. These findings elucidate a neural mechanism subserving executive control of perception and are consistent with previous literature identifying alpha‐band oscillations as the carrier frequency for communication across the visual attention system (Fries 2015), particularly within the high alpha range (D'Andrea et al. 2019; Lobier et al. 2018). While the functional connectivity measure used in the current study does not inform the direction of information flow within the network, a top‐down model of perceptual modulation is supported by previous evidence that subthreshold stimulation using transcranial magnetic stimulation of frontal (Hanning et al. 2023; Ruff et al. 2006) and parietal (Ruff et al. 2008) DAN regions enhances contrast perception and visual cortical activity in humans. Causal evidence of this top‐down model is also provided by studies in non‐human primates in which microstimulation of the frontal eye fields produces modulation of the CRF recorded across visual cortex (Ekstrom et al. 2009).

Importantly, that the relationship between DAN‐V1 high alpha‐band connectivity and *R*_max_ was observed independent of alpha‐band desynchronization suggests distinct contributions of alpha‐band connectivity and power to visual perceptual modulation. This divergence may reflect non‐attention related functions such as working memory (Erickson et al. 2019) or neural inhibition/disinhibition (Palva and Palva 2011) that have been attributed to alpha‐band modulations. DAN‐V1 functional connectivity during covert attention, however, does appear to be specific to the high alpha‐band range given the significantly lower wPLI values. While the wPLI is independent of power, it is possible that the lower band power depicted in Figure 4 could artificially deflate wPLI values. However, the lack of any meaningful relationship with *R*_max_ observed for DAN‐V1 synchronization within theta, low alpha, or beta‐band ranges suggests a unique role for the high‐alpha frequency band in this top‐down attentional modulation.

## 5 Limitations

There were limitations to the present study. Participants exhibited a ceiling effect on task performance (accuracy at even the lowest contrast levels > 95% and 92% for valid and neutral trials, respectively). While analyses of ACC and RT revealed effects of attention and stimulus contrast, an assessment of psychometric function parameters (i.e., *C*_50_ and *R*_max_) and its association with these parameters obtained from the neural response was not possible. The task also utilized only valid and neutral cue conditions. The lack of invalid cue trials restricts our assessment to attentional enhancement and limits our ability to examine alternative mechanisms underlying attention allocation including reorientation or distractor suppression. The lack of non‐target stimuli also introduces potential contamination of our visual evoked responses by target‐detection activity, though single target stimuli typically produce weak target‐evoked activity in the absence of distractors. To limit overall task duration and mitigate the impact of fatigue on task engagement, we restricted the number of contrast levels tested which can negatively impact the quality of the CRF model fit. Finally, neither inter‐stimulus nor inter‐trial interval timing was jittered in our task which limits our ability to isolate effects on intrinsic neural oscillations by introducing stimulus‐driven confounds and may have contributed to anticipatory activity leading up to stimulus presentations.

## 6 Conclusions

This study highlights the advantages of utilizing techniques with enhanced spatio‐temporal resolution to examine interactions between sensory and attentional gain properties of the human visual system, providing a comprehensive understanding of their underlying local and distributed network dynamics. MEG recordings were able to capture the non‐linear response properties of V1 to stimulus contrast, a challenge for more traditional neuroimaging methodologies. Modulation of these response properties by covert attention, consistent with established computational models of attention, was observed and the frequency‐specific, intra‐regional network communications contributing to it were identified. A similar approach could be deployed to inform debates regarding other forms of attention relying on distributed network communication such as overt or pre‐saccadic attention as well as disease states characterized by deficits in network connectivity and inattention. Future studies would also benefit from data‐driven approaches to capture more comprehensive interactions between sensory and attention networks shaping visual contrast perception outside canonical visual attention networks. Finally, the impact of age and sex on these attributes of the visual attention system should be explored in future studies utilizing larger samples with a broader age range.

## Funding

This work was supported by National Institute of Mental Health (K23 MH127389, R01 MH126951).

## Conflicts of Interest

The authors declare no conflicts of interest.

## Supporting information


**Figure S1:** Time‐frequency analysis of left and right hemisphere V1 activity to location cues directing participants to covertly shift attention to the left or right side of the visual display in preparation for the upcoming target. Time 0 s represents cue onset.

**Figure S2:** Broad‐band activity recorded from frontal and parietal regions of the DAN following presentation of the location cue (time = 0 s).

**Figure S3:** Plots depicting correlations between frontal and parietal DAN‐V1 connectivity within theta, low‐alpha, and beta frequency bands and *R*_max_ values. The relationship *R*_max_ and connectivity between V1 and primary auditory cortex (A1) was also explored within the high‐alpha frequency band.

## Data Availability

The data that support the findings of this study are available from the corresponding author upon reasonable request.
