# Supplementary figures and images for: Modulation of Visual Contrast Perception Associated With Dorsal Attention Network Connectivity Assessed by Magnetoencephalography
Source: Hum Brain Mapp. 2026 May 24;47(8):e70554. doi: 10.1002/hbm.70554 (PMC13238592; doi:10.1002/hbm.70554)

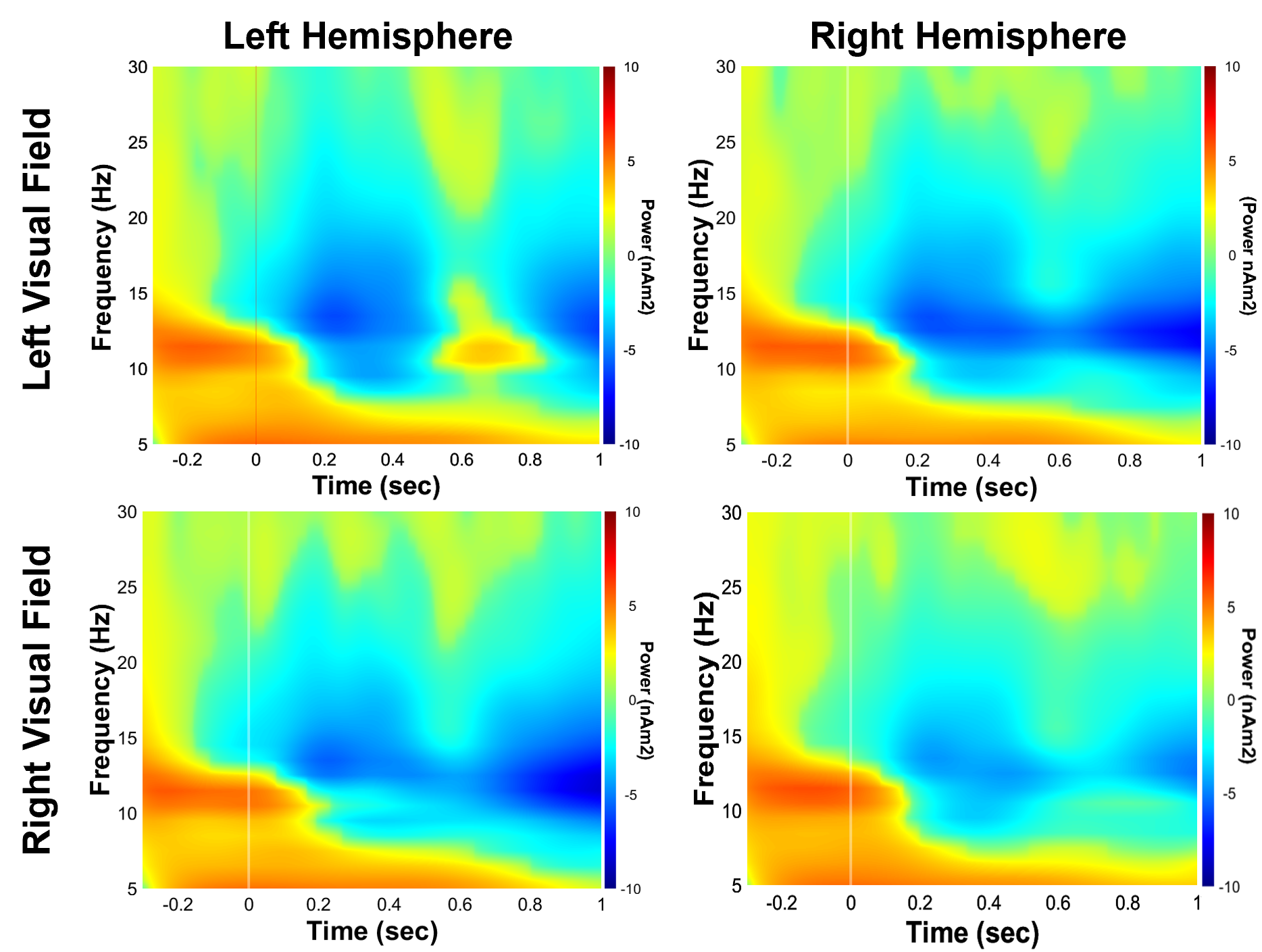

Supplement: Supplementary file 1 — Figure S1: Time‐frequency analysis of left and right hemisphere V1 activity to location cues directing participants to covertly shift attention to the left or right side of the visual display in preparation for the upcoming target. Time 0 s represents cue onset. [file HBM-47-e70554-s003.tif]

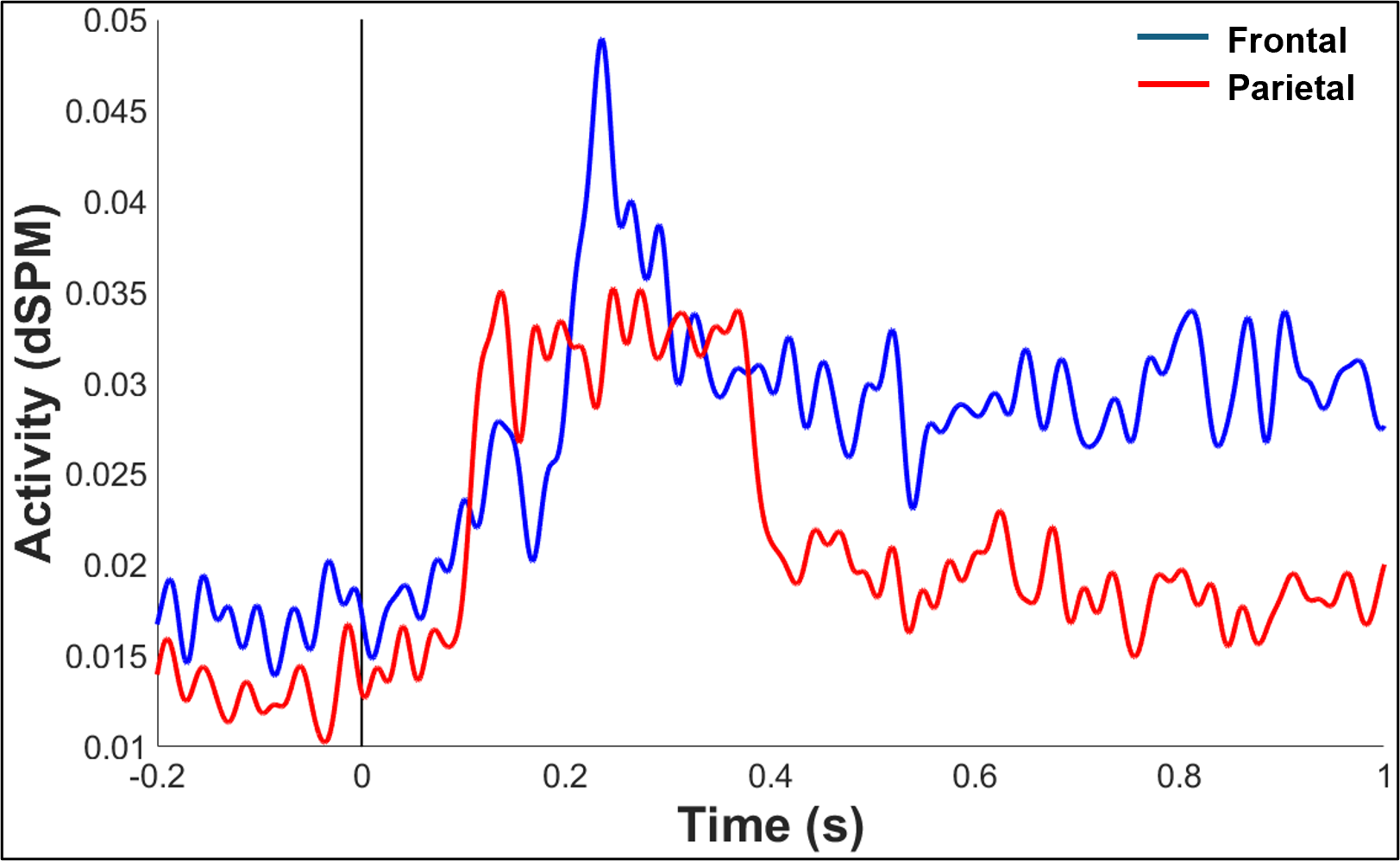

Supplement: Supplementary file 2 — Figure S2: Broad‐band activity recorded from frontal and parietal regions of the DAN following presentation of the location cue (time = 0 s). [file HBM-47-e70554-s001.tif]

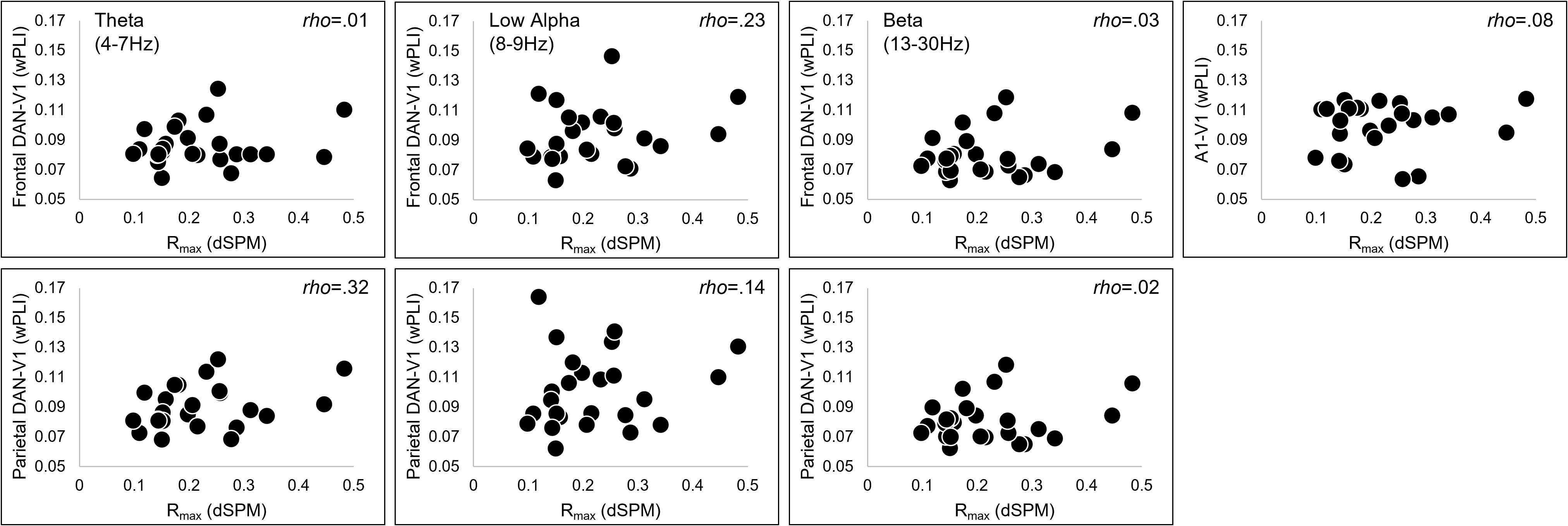

Supplement: Supplementary file 3 — Figure S3: Plots depicting correlations between frontal and parietal DAN‐V1 connectivity within theta, low‐alpha, and beta frequency bands and R max values. The relationship R max and connectivity between V1 and primary auditory cortex (A1) was also explored within the high‐alpha frequency band. [file HBM-47-e70554-s002.tif]
